# Placentas from Women with Late-Onset Preeclampsia Exhibit Increased Expression of the NLRP3 Inflammasome Machinery

**DOI:** 10.3390/biom13111644

**Published:** 2023-11-13

**Authors:** Luis M. Garcia-Puente, Oscar Fraile-Martinez, Cielo García-Montero, Julia Bujan, Juan A. De León-Luis, Coral Bravo, Patrocinio Rodríguez-Benitez, Pilar Pintado, Francisco Javier Ruiz-Labarta, Melchor Álvarez-Mon, Natalio García-Honduvilla, María J. Cancelo, Miguel A. Saez, Miguel A. Ortega

**Affiliations:** 1 Department of Medicine and Medical Specialities, Faculty of Medicine and Health Sciences, University of Alcalá, 28801 Alcalá de Henares, Spain; lmgarciapuente@uah.com (L.M.G.-P.); oscar.fraile@uah.es (O.F.-M.); cielo.gmontero@uah.com (C.G.-M.); mjulia.bujan@uah.es (J.B.); mademons@gmail.com (M.Á.-M.); natalio.garcia@uah.es (N.G.-H.); msaega1@oc.mde.es (M.A.S.); 2 Ramón y Cajal Institute of Sanitary Research (IRYCIS), 28034 Madrid, Spain; 3 Department of Public and Maternal and Child Health, School of Medicine, Complutense University of Madrid, 28040 Madrid, Spain; jaleon@ucm.es (J.A.D.L.-L.); cbravoarribas@gmail.com (C.B.); prodriguezb@senefro.org (P.R.-B.); ppintado@salud.madrid.org (P.P.); franciscojavier.ruiz@salud.madrid.org (F.J.R.-L.); 4 Department of Obstetrics and Gynecology, University Hospital Gregorio Marañón, 28009 Madrid, Spain; 5 Health Research Institute Gregorio Marañón, 28009 Madrid, Spain; 6 Department of Nephrology, University Hospital Gregorio Marañón, 28009 Madrid, Spain; 7 Immune System Diseases-Rheumatology and Internal Medicine Service, University Hospital Prince of Asturias, Networking Research Center on for Liver and Digestive Diseases (CIBEREHD), 28806 Alcalá de Henares, Spain; 8 Department of Surgery, Medical and Social Sciences, Faculty of Medicine and Health Sciences, University of Alcalá, 28801 Alcala de Henares, Spain; 9 Department of Obstetrics and Gynecology, University Hospital of Guadalajara, 19002 Guadalajara, Spain; 10 Pathological Anatomy Service, University Hospital Gómez-Ulla, 28806 Alcalá de Henares, Spain

**Keywords:** placenta, late-onset pre-eclampsia (LO-PE), NLRP3 inflammasome, caspases, interleukin 1β, interleukin 18

## Abstract

Pre-eclampsia is a harmful and potentially lethal medical condition during pregnancy clinically diagnosed by hypertension and commonly accompanied by proteinuria and multiorgan affections. According to the time of diagnosis, it is differentiated between early-onset (EO-PE) and late-onset preeclampsia (LO-PE). Despite being less dangerous and presenting distinct pathophysiological signatures, LO-PE has a greater prevalence than EO-PE, both having significant consequences on the placenta. Previous works have evidenced that exacerbated inflammation in this organ might play a potential pathogenic role in the development of pre-eclampsia, and there is some preliminary evidence that the hyperactivation of inflammasomes can be related to the altered immunoinflammatory responses observed in the placentas of these patients. However, the precise role of inflammasomes in the placentas of women with LO-PE remains to be fully understood. In this work, we have studied the gene and protein expression of the main components related to the canonical and non-canonical pathways of the inflammasome NLRP3 (NLRP3, ASC, caspase 1, caspase 5, caspase 8, interleukin 1β, and interleukin 18) in the placental tissue of women with LO-PE. Our results show a marked increase in all these components in the placentas of women who have undergone LO-PE, suggesting that NLRP3 inflammasome plays a potentially pathophysiological role in the development of this entity. Future works should aim to evaluate possible translational approaches to this dysregulation in these patients.

## 1. Introduction

Pre-eclampsia (PE) is a serious medical condition that belongs to the hypertensive disorders of pregnancy, a group of entities that affect approximately 15% of all pregnancies [1]. PE appears in around 7.5% of pregnancies [1], being clinically diagnosed by a systolic blood pressure ≥140 mm Hg or diastolic blood pressure ≥90 mm Hg on two occasions at least 4 h apart, or the shorter interval timing of systolic blood pressure ≥160 mm Hg or diastolic blood pressure ≥110 mm Hg identified after 20 weeks of gestation [2,3]. Frequently, PE can be accompanied by edemas and proteinuria, as well as fetal growth restriction or multiorgan dysfunction, which can lead to death in severe cases [4]. Despite some prophylactic measures that can be used in women at high risk of developing PE, the only definite cure for this condition is delivery, and the therapeutic alternatives in the clinical management of this condition are quite limited [5]. Because of that, a greater understanding of the pathophysiological basis of PE might aid in finding adequate alternatives and approaches to improve the clinical management of this potentially harmful disease.

The placenta is a critical organ involved in the pathogenesis of PE and multiple disorders of pregnancy [6]. Two major types of PE are identified: early-onset PE (EO-PE), also denominated placental PE, and late-onset PE (LO-PE). This classification depends on the time of the initiation of clinical symptoms, with EO-PE occurring before 34 weeks and LO-PE after 34 weeks [7]. EO-PE is associated with significant impairments in the process of placentation that lead to abnormal placental development and function, particularly affecting the remodeling of spiral arteries and trophoblast invasion, thus having more serious consequences for the mother and fetus [8]. LO-PE is, however, more common than EO-PE and appears to be ligated to maternal extrinsic factors rather than failures in the placentation process. However, compelling evidence also supports that the placentas of women with LO-PE exhibit significant and differential molecular changes when compared to EO-PE [9]. Thus, deepening the changes in and consequences of LO-PE in the placenta is critical to exploring possible consequences and promising targets or biomarkers in this group of women.

Systemic and placental inflammation seems to be a common pathophysiological signature for both EO-PE and LO-PE [10,11]. The nucleotide-binding oligomerization domain and the leucine-rich repeat-containing family pyrin domain containing 3 (NLRP3) inflammasome are multiprotein signaling platforms implicated in the induction of the inflammatory response in different tissues, which may ultimately drive to different types of cell death, such as pyroptosis and necroptosis [12]. These effects are achieved through the secretion of the proinflammatory cytokines interleukin-1β (IL-1β) and IL-18 and pyroptosis, which are, in turn, regulated by different proteins related to the NLRP3 inflammasome, such as the apoptosis-associated speck-like protein containing a CARD (ASC) and caspases 1, 5, and 8 [13]. Previous works have found that the NLRP3 inflammasome plays a crucial role in the development of physiological pregnancies, whereas it seems to be notably upregulated in different obstetric complications, including in PE [14,15,16]. However, the precise status of the NLRP3 inflammasome in the placentas of women with LO-PE remains to be further explored, especially in large cohorts. Thus, the aim of the present study is to explore the gene and protein expression of the NLRP3 inflammasome in the placental tissue and their main downstream markers (ASC, caspase 1, caspase 5, caspase 8, IL-1β, and IL-18) in a group of women with LO-PE (*n* = 68) and compare it with normal pregnancies (*n* = 43).

## 2. Patients and Methods

### 2.1. Study Design and Participants

An observational, prospective study was carried out, nested in a cohort for comparison. LOPE was diagnosed according to the criteria of the American College of Obstetricians and Gynecologists Practice Guidelines for Gestational Hypertension and Preeclampsia [7] in patients with preeclampsia who met some of the following severity criteria: systolic blood pressure (SBP) ≥160 mmHg and/or diastolic blood pressure (DBP) ≥110 mmHg confirmed at 15 min; proteinuria ≥ 2 g measured in 24 h urine or estimated by the urine protein/creatinine ratio; oliguria ≤ 500 mL/24 h or diuresis rate < 0.5 mL/kg/h for 2 h; renal failure: serum creatinine > 1.1 mg/dL, or twice the serum creatinine value in the absence of other renal disease; neurological or visual disturbances, including severe headache that does not subside with analgesics, blurred vision, diplopia, or amaurosis; acute pulmonary edema or cyanosis; pain in the epigastrium or right hypochondrium; liver dysfunction: transaminase levels elevated to twice the normal value; hematological disorders, including thrombocytopenia (<100,000 mm^3^), disseminated intravascular coagulation (DIC), or hemolysis; and placental involvement with fetal manifestations, including intrauterine growth restriction (IGR), abnormal umbilical artery Doppler results, and fetal death [17,18]. In this study, we considered the presence of a serum creatinine level greater than 1.1 mg/dL to be a criterion for the severity of preeclampsia [7]. We also included a total of 43 pregnant women free of diseases identified as healthy controls (HCs). Table 1 summarizes the main clinical features of the studied patients.

### 2.2. Sample Collection and Processing

After birth, placental biopsies were obtained. To ensure that the sample contains a variety of cotyledons, the placenta was sliced into 5 pieces in each case. These pieces were then put into a sterile tube containing Minimum Essential Medium (MEM) and 1% antibiotic/antimycotic (both from ThermoFisher Scientific, Waltham, MA, USA). Within two hours after delivery, all samples were transported by refrigeration to the laboratory. The samples were processed in a sterile environment in a laminar class II laminar flow hood (Telstar AV 30/70 Müller 220 V 50 MHz; Telstar SA Group, Terrassa, Spain). Then, histopathological and immunodetection tests were performed on the MEM samples.

Following standard techniques, placental fragments stored in the MEM were split into fragments and then fixed in F13 (60 percent ethanol, 20 percent methanol, 7 percent polyethylene glycol, and 13 percent distilled water) to remove blood cells. Blocks composed of paraffin were initially included using molds. An HM 350 S rotation microtome (Thermo Fisher Scientific, Waltham, MA, USA) was used to cut 5 µm thick slices of paraffin after it had solidified. The sections were then transferred to a hot water bath and collected on a glass slide that had been pretreated with 10% polylysine to increase the cuts’ adherence.

### 2.3. Protein Expression Studies by Immunohistochemistry

Following the established protocols [19,20], the detection of an antigen–antibody response was investigated using the ABC (avidin–biotin complex) method with peroxidase as the chromogen. The primary antibody incubation (Table 2), Abcam (Cambridge, UK), was diluted in 3% BSA and PBS and carried out throughout the course of an entire night at 4 °C. On the other hand, incubation with the secondary antibody linked to biotin and diluted in PBS was carried out for one and a half hours at room temperature. The chromogenic substrate diaminobenzidine (Kit DAB, SK-4100, Vector Laboratories, Burlingame, CA, USA), prepared just before exposure (5 mL of distilled water, two drops of buffer, four drops of DAB, and two drops of hydrogen peroxide), was then used for 60 min at room temperature (in a PBS 1:200 dilution). Brown staining is possible within this process. Sections from the same tissue were utilized in each immunohistochemical experiment as the negative controls, in which the main antibody incubation was swapped out for an incubation in PBS, a blocking solution.

### 2.4. Gene Expression Analysis Using Real-Time Quantitative PCR

Utilizing a quantitative reverse transcription polymerase chain reaction (RT-qPCR), the expression of the target genes was investigated. Each sample’s cDNA concentration (Thermo Fisher Scientific) was measured. The guanidine–phenol–chloroform isothiocyanate technique was used to extract the RNA [21], and the Primer-BLAST tool and the Auto-Dimer program were used to create the primers that were used [22,23]. We performed qPCR using the StepOnePlusTM equipment and the relative standard curve approach.

In total, 5 µL of each sample was combined with 10 µL of the intercalating agent iQTM SYBR^®^ Green Supermix (Bio-Rad Laboratories, Hercules, CA, USA), 1 µL of the forward primer, 1 µL of the reverse primer, and 3 µL of DNase and RNase-free water after being diluted with nuclease-free water. A MicroAmp^®^ 96-well plate (Applied Biosystems-Life Technologies, Foster City, CA, USA) was used to evaluate the 20 µL solutions. Glyceraldehyde 3-phosphate dehydrogenase (GAPDH; Table 3), a housekeeping gene, was used to standardize the final data and compare them to it. The standard curve was employed to interpolate the data collected for each gene. The standard curve was tested twice, the samples were tested three times, and the negative controls were placed in the other two wells.

### 2.5. Statistical Analysis

GraphPad Prism^®^ 6.0 was utilized for the statistical analysis, and a Mann–Whitney U test was performed. Interquartile range (IQR) and the median are used to express data. Values of *p* 0.05 (*), *p* 0.01 (**), and *p* 0.001 (***) were used to determine the significance. Five counts were applied at random to tissue slices to count the immunopositive cells, eliminating cells that did not cross the designated demarcation lines. According to the anatomopathological criteria outlined by previous studies, patients were considered positive when the test sample score for each subject was greater than or equal to 5% of the total through the immunoreactive score (ISR score) [24]. Two separate histologists (M.A.O. and M.A.S.), who were blinded to the outcome measure, evaluated the tissue’s immunostaining. The cuts were examined using a Carl Zeiss Axiophot (Jena, Germany) optical microscope.

## 3. Results

### 3.1. The Placentas of Women with Late-Onset Preeclampsia Show Enhanced Gene and Protein Expression of NLRP3 and ASC

**Figure 1:**
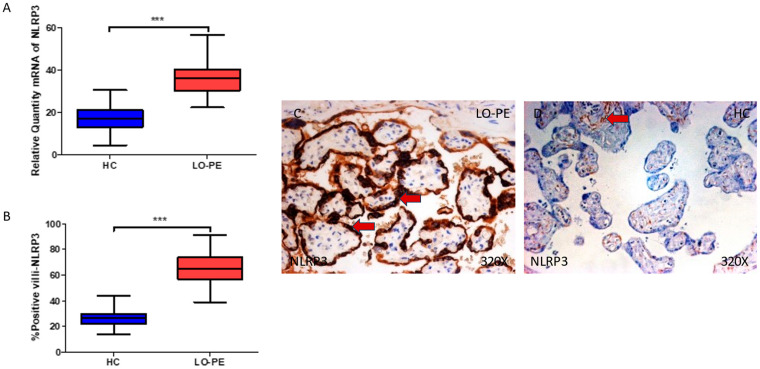
(**A**) NLRP3 mRNA expression in women with LO-PE and HC. (**B**) IRS scores for NLRP3 expression in the placental villi of the LO-PE and HC group. (**C**,**D**) Images showing immunostaining for NLRP3 in the placental villi of the LO-PE and HC. *p* < 0.001 (***). Red arrows: Tissue expression of NLRP3 was strongly evidenced in the syncytiotrophoblast layer of women with LO-PE, whereas for HC, it is more limited to the cytotrophoblast and other cells located in the inner of placental villi.

Firstly, our findings demonstrate a statistically significant increase in NLRP3 gene expression in the placental tissue of pregnant women who have LO-PE (*** *p* < 0.0001; LO-PE = 36.309 [22.361–56.361], HC = 16.982 [4.651–30.562], Figure 1A). Histological analysis of the placental villi showed that the chorionic villi of women with LO-PE showed a significant increase in the protein expression of NLRP3, (*** *p* < 0.0001; LO-PE = 65.000 [39.000–91.000], HC = 27.000 [14.000–44.000], Figure 1B). The tissue expression of NLRP3 was strongly evidenced throughout the placental villi of women affected by LO-PE in comparison to HC, particularly in the syncytiotrophoblast layer (Figure 1C,D).

**Figure 2:**
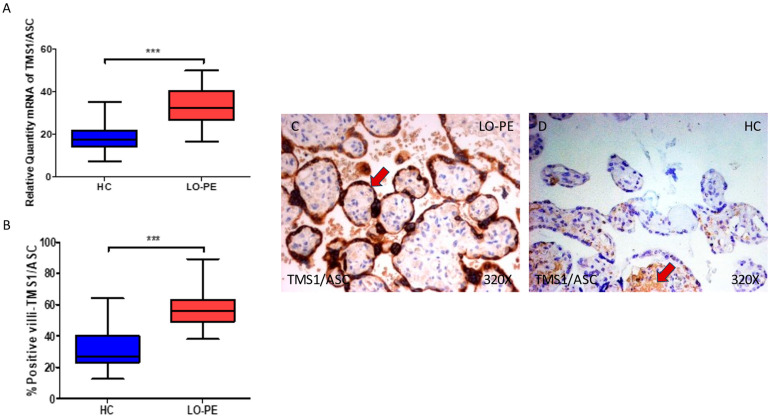
(**A**) ASC mRNA expression in women with LO-PE and HC. (**B**) IRS scores for ASC expression in the placental villi of the LO-PE and HC group. (**C**,**D**) Images showing immunostaining for ASC in the placental villi of the LO-PE and HC. *p* < 0.001 (***). In red arrows, it can be observed that expression of this component in the placentas of women with LO-PE is mainly found in the syncytiotrophoblast layer, whereas for HC, it is observed in cytotrophoblast and the other cells present in the inner of the placental villi.

In parallel, we observed a significant increase in ASC gene expression in the placental tissue of pregnant women who have LO-PE (*** *p* < 0.0001; LO-PE = 32.284 [16.562–49.768], HC = 17.562 [7.317–35.326], Figure 2A). Histological analysis of the placental villi showed that the chorionic villi of women with LO-PE showed a significant increase in the protein expression of ASC (*** *p* < 0.0001; LO-PE = 56.000 [38.000–89.000], HC = 27.000 [12.500–64.000], Figure 2B). The tissue expression of ASC was strongly evidenced throughout the placental villi of women affected by LO-PE, particularly in the syncytiotrophoblast layer in comparison to HC, in which a moderate expression of this marker is observed in the cytotrophoblast and other cells located in the inner layer of the placental villi (Figure 2C,D).

### 3.2. Women with Late-Onset Preeclampsia Display an Increased Expression of Caspase 1, Caspase 5, and Caspase 8

**Figure 3:**
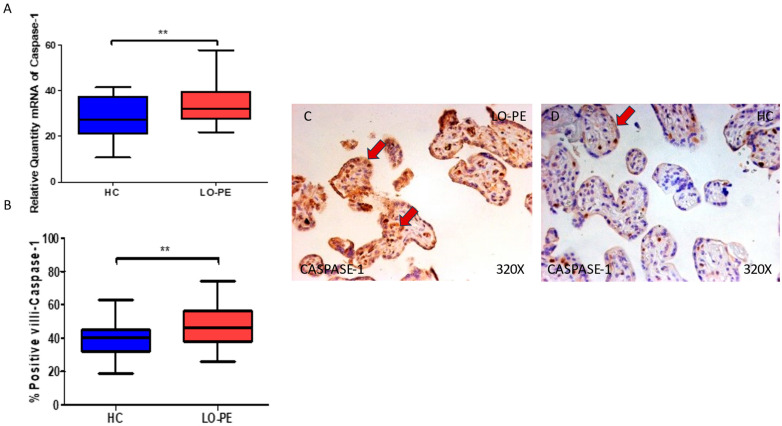
(**A**) Caspase 1 mRNA expression in women with LO-PE and HC. (**B**) IRS scores for caspase 1 expression in the placental villi of the LO-PE and HC group. (**C**,**D**) Images showing immunostaining for caspase 1 in the placental villi of the LO-PE and HC. *p* < 0.01 (**). In red arrows, the expression of caspase 1 in the syncytiotrophoblast layer and also in the cytotrophoblast and other cells in the inner layer of the placental villi in women with LO-PE is highlighted, whereas for HC, it is more located in the syncytiotrophoblast layer.

We then considered the study of caspases 1, 5, and 8 in the placental tissue of women with LO-PE. Our work supports a statistically significant increase in caspase 1 gene expression in the placental tissue of pregnant women who have LO-PE (** *p* = 0.0019; LO-PE = 32.041 [21.950–57.951], HC = 27.562 [10.685–41.562], Figure 3A). Histological analysis of the placental villi showed that the chorionic villi of women with LO-PE showed a significant increase in the protein expression of caspase 1, (** *p* = 0.0018; LO-PE = 46.000 [26.000–74.000], HC = 40.000 [19.000–63.000], Figure 3B). Caspase 1 was prominently expressed in the syncytiotrophoblast layer in women with LO-PE, but also in the cytotrophoblast and other cells in the inner layer of the placental villi (Figure 3C,D).

**Figure 4:**
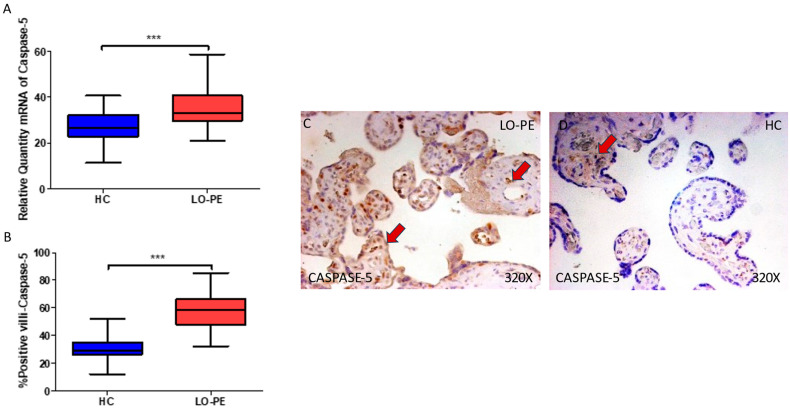
(**A**) Caspase 5 mRNA expression in women with LO-PE and HC. (**B**) IRS scores for caspase 5 expression in the placental villi of the LO-PE and HC group. (**C**,**D**) Images showing immunostaining for caspase 5 in the placental villi of the LO-PE and HC. *p* < 0.001 (***). In red arrows, caspase 5 expression is observed throughout the placental villi of women affected by LO-PE in comparison to HC, in which this marker can be slightly observed in the cytotrophoblasts and other cells in the inner layer of the placental villi.

Concomitantly, we observed a significant increase in caspase 5 gene expression in the placental tissue of pregnant women who have LO-PE (*** *p* < 0.0001; LO-PE = 58.500 [32.000–85.000], HC = 26.546 [11.453–40.619], Figure 4A). Histological analysis of the placental villi showed that the chorionic villi of women with LO-PE showed a significant increase in the protein expression of caspase 5 (*** *p* < 0.0001; LO-PE = 58.500 [32.000–85.000], HC = 29.000 [12.000–52.000], Figure 4B). The tissue expression of caspase 5 was strongly displayed throughout the placental villi of women affected by LO-PE in comparison to HC, in which this marker can be slightly observed in the cytotrophoblasts and other cells in the inner layer of the placental villi (Figure 4C,D).

**Figure 5:**
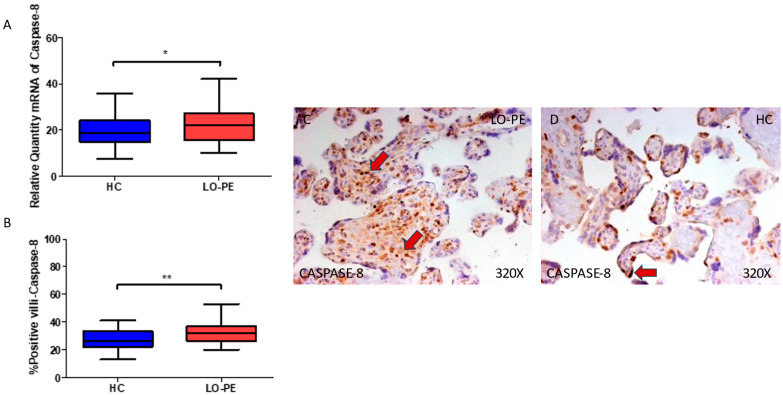
(**A**) Caspase 8 mRNA expression in women with LO-PE and HC. (**B**) IRS scores for caspase 8 expression in the placental villi of the LO-PE and HC group. (**C**,**D**) Images showing immunostaining for caspase 8 in the placental villi of the LO-PE and HC. *p* < 0.01 (**); *p* < 0.05 (*). Red arrows: caspase 8 expression is evident in the cytotrophoblasts and other cells located in the inner of the placental villi of women affected by LO-PE in comparison to HC, in which the expression of this marker can be differentially observed in syncytiotrophoblasts.

Finally, regarding caspase 8, we observed an enhanced gene expression of this component in the placental tissue of women with LO-PE (* *p* = 0.0370; LO-PE = 22.019 [10.156–42.006], HC = 18.652 [7.515–35.616], Figure 5A). Histological analysis of the placental villi showed that the chorionic villi of women with LO-PE showed a significant increase in the protein expression of caspase 8 (** *p* = 0.0014; LO-PE = 32.000 [20.000–53.000], HC = 26.000 [13.000–41.000] Figure 5B). The tissue expression of caspase 8 was more evident in the cytotrophoblasts and other cells located in the inner of the placental villi of women affected by LO-PE in comparison to HC, in which the expression of this marker can be differentially observed in syncytiotrophoblasts (Figure 5C,D).

### 3.3. The Placental Tissue of Women with Late-Onset Preeclampsia Exhibits Augmented Expression of IL-1β and IL-18

**Figure 6:**
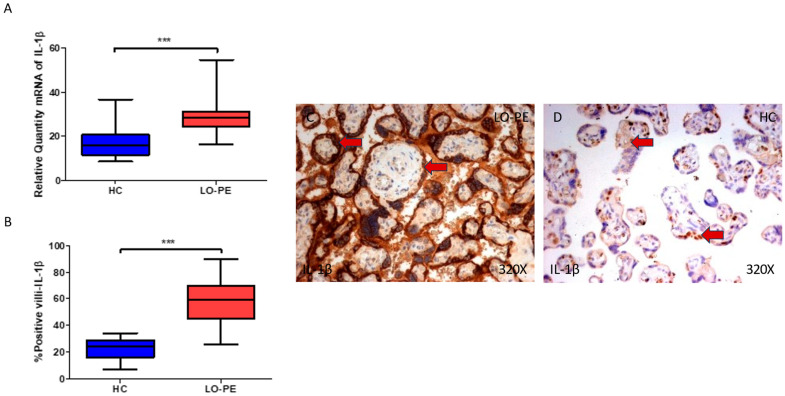
(**A**) IL-1β mRNA expression in women with LO-PE and HC. (**B**) IRS scores for IL-1β expression in the placental villi of the LO-PE and HC group. (**C**,**D**) Images showing immunostaining for IL-1β in the placental villi of the LO-PE and HC. *p* < 0.001 (***). Red arrow: IL-1β is highly expressed in the syncytiotrophoblast layer of the placentas of women affected by LO-PE in comparison to HC, in which the expression of this marker is slight and almost limited to some syncytiotrophoblasts and cytotrophoblasts.

Finally, we explored IL-1β and IL-18 in the placental tissue of women with LO-PE. We observed that the IL-1β gene expression in the placental tissue of pregnant women who have LO-PE is notably increased (*** *p* < 0.0001; LO-PE = 28.361 [16.306–54.697], HC = 16.016 [8.651–36.652], Figure 6A). Histological analysis of the placental villi showed that the chorionic villi of women with LO-PE showed a significant increase in the protein expression of caspase 1 (*** *p* < 0.0001; LO-PE = 59.500 [26.000–90.000], HC = 24.000 [7.000–34.000], Figure 6B). The tissue expression of IL-1β was highly expressed in the syncytiotrophoblast layer of the placentas of women with LO-PE in comparison to HC, in which the expression of this marker is slight and almost limited to some syncytiotrophoblasts and cytotrophoblasts (Figure 6C,D).

**Figure 7:**
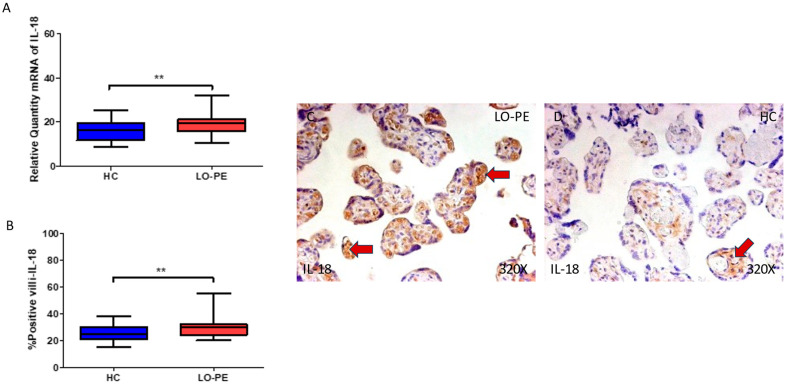
(**A**) Caspase 1 mRNA expression in women with LO-PE and HC. (**B**) IRS scores for caspase 1 expression in the placental villi of the LO-PE and HC group. (**C**,**D**) Images showing immunostaining for caspase 1 in the placental villi of the LO-PE and HC. *p* < 0.01 (**). Red arrows: IL-18 expression was strongly displayed throughout the placental villi of women affected by LO-PE in comparison to HC, in which a moderate expression of this cytokine can be observed in cytotrophoblast and other cells located in the inner of the placental villi.

Regarding IL-18, we report that the gene expression in the placental tissue of pregnant women who have LO-PE is notably increased (** *p* = 0.0013; LO-PE = 19.568 [10.326–32.032], HC = 16.236 [8.616–25.321], Figure 7A). Histological analysis of the placental villi showed that the chorionic villi of women with LO-PE showed a significant increase in the protein expression of caspase 1 (*** *p* < 0.0001; LO-PE = 30.000 [20.000–55.000], HC = 25.000 [15.000–38.000] Figure 7B). The tissue expression of IL-18 was strongly displayed throughout the placental villi of women affected by LO-PE in comparison to HC, in which a moderate expression of this cytokine can be observed in cytotrophoblast and other cells located in the inner of the placental villi (Figure 7C,D).

## 4. Discussion

LO-PE is a type of obstetric complication associated with unique and differential alterations in the placenta. It is hypothesized that LO-PE is secondary to intraplacental (intervillous) malperfusion related to mechanical restrictions and an inability of the cardiovascular system to meet the increased metabolic demands of the fetoplacental unit [11,25]. Consequently, different responses in this tissue are conducted, including altered mitochondrial structure and function, oxidative stress, cell death, and inflammation [26]. In this study, we have found that LO-PE is associated with an exacerbated inflammasome activation in the placenta, evidenced by the increased gene and protein expression of NLRP3, ASC, caspase 1, caspase 5, caspase 8, IL-1β, and IL-18.

The NLRP3 inflammasome is a pivotal mechanism of the innate immune system activated in response to microbial infection and cellular damage [27]. The expression of the NLRP3 inflammasome in the placenta fulfills multiple roles in physiological pregnancies and under different obstetric complications [15]. The placenta is an organ that express the inflammasome(s) constitutively [28]. In vitro studies have defined that trophoblasts and other placental cells expressed NLRP1, NLRP3, NLRC4, and NLRP7 inflammasomes in the first trimester of pregnancy, and some of them also at term, especially in association with parturition [15,29]. The peripheral immune cells of pregnant women also exhibit enhanced expression of the NLRP3 inflammasome as pregnancy progress [30]. The placental tissue and peripheral immune cells of women with PE present augmented levels of NLRP3, mainly attributed to the increased release of damage-associated molecular patterns (DAMPs) like cholesterol crystals, extracellular DNA, high-mobility group box 1 (HMGB1), extracellular cell debris, advanced glycation end-products (AGEs), and free fatty acids [31]. Likewise, uric acid is another DAMP with a crucial role in the pathogenesis of PE noted by an increase in the enzyme xanthine oxidase in the placenta that promotes the conversion of xanthine into uric acid with the generation of a superoxide anion that enhances oxidative stress, inflammation, and endothelial dysfunction [32]. Previous works have shown that the deleterious effects of uric acid are partly related to the exacerbated hyperactivation of the NLRP3 inflammasome in pre-eclamptic women [33,34], demonstrating the relevance of the activation of the NLRP3 inflammasome by different DAMPs in the development of PE.

NLRP3 hyperactivation seems to be regulated by canonical and non-canonical pathways. This occurs in a two-step process: Step 1 initiates after the recognition of certain DAMPs and pathogen-associated molecular patterns (PAMPs), which after binding to their receptors promote the translocation of the nuclear factor kappa beta (NF-κB) into the nucleus, activating the transcription of NLRP3, caspase 1, IL-1β, and IL-18. Then, step 2 starts with the sensing of danger signals by NLRP3 that trigger the activation of NLRP3 inflammasome through potassium K+ efflux, inducing NLRP3 and ASC oligomerization and assembly [13]. ASC is an adaptor protein of different inflammasomes, including NLRP3 [35]. The ASC protein is broadly expressed in a plethora of cells and tissues, including the trophoblasts cells in the placenta [36]. Indeed, then an interaction between NLRP3 and ASC is essential to form functional NLRP3 inflammasomes and to mediate caspase 1 activation [37]. Then, NLRP3 inflammasome recruits and activates procaspase 1. Caspase 1 is responsible for cleaving pro-IL-1β, pro-IL-18, and gasdermin D (GSDMD) at D116, D36, and D275, respectively [38]. GSDMD is responsible for creating a transmembrane pore that disrupts ion and water equilibrium and secretes IL-1β and IL-18, two major inflammatory cytokines with pleiotropic functions [39]. Both IL-1β and IL-18 are members of the IL-1 family, binding into receptors of the IL-1 receptor family. In general terms, the actions of these cytokines are crucial in the orchestration of inflammatory responses from the innate and adaptive immunity, metabolism, and modulation of plenty biological functions in multiple tissues [13]. However, prior works have also found significant variations in their signaling platforms, differentially modulating multiple cell populations and biological processes [40]. In the placenta, an upregulation of these cytokines has been related to different obstetric complications [41,42,43], and it is well documented that abnormal levels of these cytokines are elevated in the setting of hypertension [44]. The increased expression of NLRP3, ASC, caspase 1, IL-1β, and IL-18 in the placenta of women with LO-PE might be indicating that the increased presence of DAMPs activates NLRP3, which interacts with ASC and caspase 1, that in turn leads to the maturation and secretion of IL-1β and IL-18, evidencing the relevance of the NLRP3 inflammasome in this tissue. Thus, in agreement with previous works, our results seem to support the potential pathophysiological role of the NLRP3 inflammasome in the development and progression of PE [31,45]. The precise consequences for the exacerbated activation of the inflammasome in the placentas of women with LO-PE remain to be deeply explored.

On the other hand, caspase 5 and caspase 8 are also major inducers of IL-1ß and IL-18 secretion and release, although they act by non-canonical pathways. In the case of caspase 5, previous works have described that this protein can directly lead to the release of IL-1β and IL-18 after exposure to DAMPs like extracellular heme and pathogen-associated molecular patterns (PAMPs) like lipopolysaccharides (LPS) [46,47]. Indeed, caspase 4/5 can act through two major pathways: (a) by cleaving GSDMD and creating pores in the cell membrane to induce pyroptotic cell death, or through the activation of the NLRP3 inflammasome [48]. Caspase 8 is also able to promote different types of cell death, including pyroptosis and the release of IL-1β and IL-18 by mediating the activation of the inflammasome in response to the activation of cell surface death receptors (DRs) [49]. In more detail, caspase 8 can act either to induce apoptosis or in requirement from the optimal expression of NLRP3 and pro IL-1β possibly due to their role in NF-κB activation [50]. An altered expression of caspase 8 in the placentas of women with different obstetric complications like preterm labor and PE has been reported in past works [51,52]; although, to our best knowledge, the expression of caspase 5 in the placental tissue has not been evaluated in pregnancy diseases. Interestingly, we report that despite both caspases being elevated in our study, the protein and gene expression of caspase 5 is more marked than caspase 8, evidencing the need to further efforts for understanding the possible actions of caspase 5 in the placentas of women with LO-PE. Our results support the potential pathophysiological role of caspase 5 and 8, both related to the non-canonical actions of the inflammasome, although additional studies are warranted in this sense.

## 5. Conclusions

**Figure 8:**
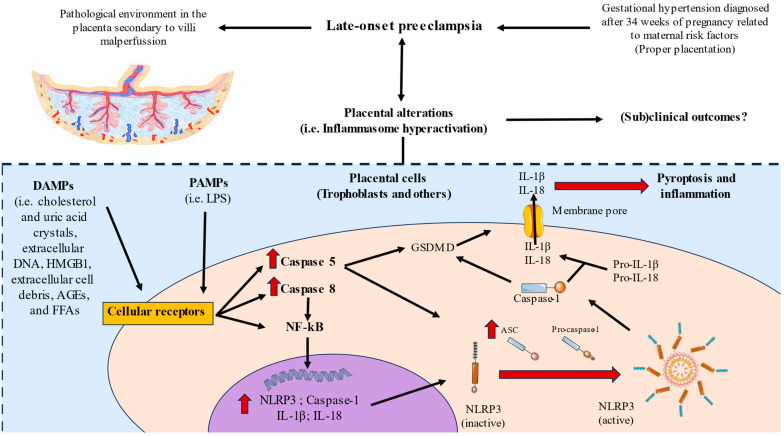
Graphical abstract of the results observed in our study.

In this study, we have observed a marked increase in the gene and protein expression of NLRP3, ASC, caspases1, 5, and 8, as well as IL-1β and IL-18, suggesting that these components play a potentially pathophysiological role in the placental tissue of women who have undergone LO-PE (Figure 8). Future works should be directed towards evaluating possible translational approaches of this dysregulation in these patients.

## Figures and Tables

**Table 1:** Clinical features of the included patients. *p* 0.05 (*), and *p* 0.001 (***).

	HC (*n* = 43)	LO-PE (*n* = 68)	*p*-Value
Maternal Age (years) Mean ± SD	31,348 ± 5117	29,015 ± 4816	* *p* = 0.0154
Nulliparous (%) Total number	14 (32.56)	53 (77.94)	*** *p* < 0.0001
Gestation (weeks)	39,069 ± 1486	38,627 ± 1434	NS
C-Section (%) Total number	8 (18.60)	15 (22.06)	NS
Placental Weight (g)	500,977 ± 65,331	370,254 ± 61,647	*** *p* < 0.0001

**Table 2:** Primary and secondary antibodies and their dilutions.

Antigen	Species	Dilution	Provider	Protocol Specifications
NLRP3	Rabbit Monoclonal	1:500	Abcam (ab263,899)	10 mM Sodium citrate pH = 6, before incubation with blocking solution
ASC	Rabbit monoclonal	1:250	Abcam (ab283,684)	100% Triton 0.1% in PBS for 10 min, before incubation with blocking solution
Caspase 1	Rabbit Polyclonal	1:500	Abcam (ab62,698)	EDTA pH = 9, before incubation with blocking solution
Caspase 5	Rabbit monoclonal	1:100	Abcam (ab40,887)	10 mM Sodium citrate pH = 6, before incubation with blocking solution
Caspase 8	Rabbit polyclonal	1:250	Abcam (ab25,901)	100% Triton 0.1% in PBS for 10 min, before incubation with blocking solution
IL-1β	Rabbit recombinant multiclonal	1:50	Abcam (ab283,818)	Not Specifications
IL-18	Rabbit monoclonal	1:50	Abcam (ab243,091)	Not Specifications
IgG (Rabbit)	Mouse	1:1000	Sigma-Aldrich (RG96/B5283)	Not Specifications

**Table 3:** Primers used for RT-qPCR: sequences and binding temperatures (Temp).

GENE	SEQUENCE Fwd (5′→3′)	SEQUENCE Rev (5′→3′)	Temp
TBP	TGCACAGGAGCCAAGAGTGAA	CACATCACAGCTCCCCACCA	60 °C
NLRP3	GCTGGCATCTGGATGAGGAA	GTGTGTCCTGAGCCATGGAA	61 °C
ASC	ATCCAGGCCCCTCCTCAG	AGAGCTTCCGCATCTTGCTT	60 °C
Caspase 1	GAAAAGCCATGGCCGACAAG	GCTGTCAGAGGTCTTGTGCT	57 °C
Caspase 5	TGTTAGCTATGGCTGAAGACAGT	TTGATGAGCCACGCGATTCT	58 °C
Caspase 8	GTCTGTACCTTTCTGGCGGA	CTCAGGCTCTGGCAAAGTGA	60 °C
IL-1β	AGCCATGGCAGAAGTACCTG	TGAAGCCCTTGCTGTAGTGG	60 °C
IL-18	GCTGAAGATGATGAAAACCTGGA	GAGGCCGATTTCCTTGGTCA	59 °C

## Data Availability

The data used to support the findings of the present study are available from the corresponding author upon request.
